# Screening for psychological distress among workaholic engineers

**DOI:** 10.1192/j.eurpsy.2025.1590

**Published:** 2025-08-26

**Authors:** A. Hrairi, N. Rmadi, M. Hajji, F. Dhouib, N. Kotti, M. Hajjaji, K. Jmal Hammami

**Affiliations:** 1 Occupational medicine department, Hedi chaker university hospital, University of Sfax; 2 Family medicine department, University of Sfax, Sfax, Tunisia

## Abstract

**Introduction:**

Several factors can threaten the mental health of engineers and seem to lead to anxiety and depressive disorders. Workaholism is an emerging phenomenon that has been the topic of much debate on its impact on workers’ mental health.

**Objectives:**

Determining the prevalence of workaholism among women and men engineers and screening for psychological distress among the workaholic engineers with a focus on gender differences.

**Methods:**

This study is a descriptive-cross sectional analysis conducted on active engineers for one month. Data were collected through an online questionnaire, including socio-professional data, the WART (Work Addiction Risk Test) and the Hospital Anxiety and Depression scale.

**Results:**

Our population consisted of 45 women and 62 men engineers with an average age of 28.62± 4.3 years and 29.61± 4.5 years respectively. A high risk of workaholism was present among 42.2% and 41.9% of women and men respectively.

Among workaholic engineers, anxiety and depression were present in 73.1% and 46.2% of cases respectively among men and in 78.9% and 42.1% of cases respectively among women.

Workaholic engineers women were likely to have anxiety (p=0.000) and reproach from their families for excessive professional commitment (p=0.007).

Among engineers men, associations were found between workaholism and anxiety (p=0.000), depression (p=0.024), the use of psychotropic medication (p=0.013), a job satisfaction less than 4/10 (p=0.024) and facing reproach from their families for excessive professional commitment (p=0.032).

Workaholism among both women and men engineers was negatively correlated with sports activities (p=0.006, p=0.042).

**Conclusions:**

Workaholism is a significant phenomenon among engineers that can lead to anxiety and depression disorders. Therefore, the detection of early signs of workaholism and its associated symptoms seems essential among this vulnerable population in order to prevent its psychological impact.

**Disclosure of Interest:**

None Declared

